# Array-based DNA methylation profiling of primary lymphomas of the central nervous system

**DOI:** 10.1186/1471-2407-9-455

**Published:** 2009-12-21

**Authors:** Julia Richter, Ole Ammerpohl, José I Martín-Subero, Manuel Montesinos-Rongen, Marina Bibikova, Eliza Wickham-Garcia, Otmar D Wiestler, Martina Deckert, Reiner Siebert

**Affiliations:** 1 Institute of Human Genetics, University Hospital Schleswig-Holstein Campus Kiel/Christian-Albrechts University Kiel, Germany; 2 Department of Neuropathology, University of Cologne, Cologne, Germany; 3 Illumina Inc., San Diego, CA, USA; 4 Molecular Neuro-Oncology, German Cancer Research Center (DKFZ), Heidelberg, Germany

## Abstract

**Background:**

Although primary lymphomas of the central nervous system (PCNSL) and extracerebral diffuse large B-cell lymphoma (DLBCL) cannot be distinguished histologically, it is still a matter of debate whether PCNSL differ from systemic DLBCL with respect to their molecular features and pathogenesis. Analysis of the DNA methylation pattern might provide further data distinguishing these entities at a molecular level.

**Methods:**

Using an array-based technology we have assessed the DNA methylation status of 1,505 individual CpG loci in five PCNSL and compared the results to DNA methylation profiles of 49 DLBCL and ten hematopoietic controls.

**Results:**

We identified 194 genes differentially methylated between PCNSL and normal controls. Interestingly, Polycomb target genes and genes with promoters showing a high CpG content were significantly enriched in the group of genes hypermethylated in PCNSL. However, PCNSL and systemic DLBCL did not differ in their methylation pattern.

**Conclusions:**

Based on the data presented here, PCNSL and DLBCL do not differ in their DNA methylation pattern. Thus, DNA methylation analysis does not support a separation of PCNSL and DLBCL into individual entities. However, PCNSL and DLBCL differ in their DNA methylation pattern from non- malignant controls.

## Background

Primary lymphomas of the central nervous system (PCNSL) are highly malignant B-cell lymphomas confined to the central nervous system (CNS) with a poor prognosis [1]. They are considered as separate entity within the updated WHO classification [1], although they cannot be distinguished histologically and immunophenotypically from extracerebral diffuse large B-cell lymphoma (DLBCL). However, based on the remarkably worse clinical course and prognosis it is still a matter of debate whether PCNSL differ from systemic DLBCL with respect to their molecular features and pathogenesis. Interphase cytogenetic and molecular genetic studies have shown that PCNSL share a variety of features with systemic DLBCL. These include rearranged immunoglobulin (*IG*) gene segments with evidence for ongoing somatic hypermutation, aberrant somatic hypermutation of non-*IG *genes, translocations affecting the *IG *and *BCL6 *genes, gains in chromosome band 18q21, and mutations of the *PRDM1 *gene [2]. With respect to their gene expression profile PCNSL segregate along the spectrum of systemic DLBCL including the ABC- and GCB-types of DLBCL [3,4].

Epigenetic silencing of functionally important genes by DNA methylation may also contribute to PCNSL development [5-10]. By studying DNA methylation of 14 tumor suppressor genes in 25 PCNSL using methylation-specific PCR, Chu et al. [5] demonstrated that all PCNSL had methylated at least two of the genes studied. Although these findings suggest DNA methylation of tumor suppressor genes to be a common event in PCNSL, previous studies on DNA methylation have been limited to a rather small number of a few selected genes. The recent availability of array-based techniques offers the opportunity for a more comprehensive DNA methylation profiling [11,12]. Here, a series of PCNSL was studied for DNA methylation of 1,505 CpG sites from 807 selected genes including a significant number of genes relevant for tumorigenesis. By comparing DNA methylation profiles of PCNSL to those recently obtained for normal hematopoietic controls and 49 systemic DLBCL [12], we identified 194 genes to be differentially methylated between PCNSL and normal controls. Four genes were putatively differentially methylated between PCNSL and systemic DLBCL. Based on the DNA methylation pattern these lymphoma entities did not segregate suggesting DNA methylation pattern of PCNSL and systemic DLBCL to be highly comparable.

## Methods

### DNA extraction and samples

DNA samples from five tumors diagnosed as PCNSL (two of ABC- and three of GCB-subtype, all from female patients) as described recently [3] and classified according to the WHO classification 2008 [1] were subjected to array-based DNA methylation profiling. Systemic lymphoma manifestation was excluded by extensive staging. All studies were approved by local ethics committees. Informed consent was provided according to the Declaration of Helsinki. DNA extraction was performed as described previously [13]. The DNA samples of 49 systemic DLBCL and of 10 normal controls have been described in detail recently [12,14]. The tumor cell content of DLBCL samples was >70% as verified by a panel of experienced pathologists.

### DNA methylation profiling using universal BeadArrays

DNA methylation analyses were performed using the GoldenGate Methylation Cancer Panel I (Illumina Inc., San Diego, CA) as described previously [12]. The array allows assaying 1,505 CpG sites from 807 selected genes, which include numerous genes relevant for tumorigenesis including oncogenes, tumor suppressor genes, and genes involved in metastasis, differentiation, cell cycle control, and apoptosis. The complete dataset is provided as supplementary information (Additional file 1). The reproducibility and accuracy of the GoldenGate Cancer Panel I based DNA methylation analysis has been demonstrated extensively [11,12].

### Analyses of the DNA methylation data obtained by the GoldenGate Methylation Cancer Panel I

DNA methylation profiling data from 10 hematopoietic controls (analysed in replicates) and 49 systemic DLBCL obtained recently with the same platform served for comparison and have been reanalyzed in this study [12]. As detailed in Additional file 2, normal controls contained two samples of tonsillar germinal center B-cells, two normal peripheral blood samples, and six lymphoblastoid cell lines. Systemic DLBCL were classified as non molecular Burkitt lymphoma (non-mBL) by gene expression profiling and included 29 ABC and 20 GCB-type DLBCL [12,14]. Identification of imprinted CpGs and gender-specifically methylated CpGs on chromosome X has been performed as described recently [12]. Using BeadStudio software (ver.3, Illumina Inc., San Diego, CA) agglomerative hierarchical clustering was performed in PCNSL, systemic DLBCL, and normal hematopoietic controls excluding CpG loci located in imprinted genes and X-chromosomal genes with gender-specific methylation (1,284 CpGs corresponding to 716 genes were included for further analysis). The statistical analyses used to define the different DNA methylation subgroups have been detailed recently [12]. The data obtained from the arrays were not normalized. In addition, differential methylation analysis (DMA) has also been performed using the BeadStudio Software. CpG loci with calculated DiffScores below -30 or above 30 (corresponding to a p-value of p > 0.001, based on a t-statistic) simultaneously showing an absolute DeltaBeta value above 0.3 (corresponding to a difference of 30% in the DNA methylation level) were considered as differentially methylated. The global DNA methylation data per case shows a bimodal distribution in which beta values < 0.25 defines the unmethylated CpGs and beta values> 0.75 the methylated CpGs [12]. Thus, beta values below 0.25 and above 0.75 were selected as threshold-values to define unmethylated and methylated CpG loci for further analysis.

### Enrichment for PcG-marks and promoter classes in different DNA methylation groups

Identification of Polycomb target genes and promoter classification into promoters with high (HCP), intermediate (ICP), and low (LCP) CpG content respectively, are based on recent publications [15-17]. Annotation lists of Polycomb group (PcG) marks and promoter classes were compared with the genes analyzed for methylation via gene symbol or locuslink ID. Proportions of genes in the different methylation groups were compared with respect to PcG-marks and promoter classes as described previously [12].

### Principal component analysis (PCA)

PCA has been performed using the Omics Explorer, Version 2.0 Beta (Qlucore AB, Lund, Sweden).

### Gene ontology

The "Gene Annotation Tool to Help Explain Relationships" (GATHER; http://gather.genome.duke.edu) has been used to determine the enrichment of individual ontology terms in the group of genes differentially methylated in PCNSL and normal controls (with respect to the overall composition of the GoldenGate array). Ontology terms with an at least twofold increase in numbers of genes between the tested group and the array containing at least five individual genes, were tested for significance of enrichment by performing Fisher's exact test.

### Statistics

Fisher's exact test and Mann-Whitney test were performed using GraphPad Prism version 4.02 for Windows, GraphPad Software, San Diego, CA.

## Results

### Array-based DNA methylation profiling of PCNSL

The BeadArray technology [11,18] was applied to perform quantitative DNA methylation analyses of 1,505 individual CpGs corresponding to 807 genes relevant for tumorigenesis in five PCNSL. Results were compared to those recently obtained for ten hematopoietic controls and 49 systemic DLBCL [12]. Genes known to be imprinted and X-chromosomal genes with gender-specific methylation were excluded from the analysis, as they are partially methylated under physiological conditions and might represent a confounding variable to classify cases according to their DNA methylation profile. Thus, a total of 1,284 CpGs corresponding to 716 genes entered the analysis.

### Identification of genes differentially methylated between PCNSL and hematopoietic controls

**Figure 1:**
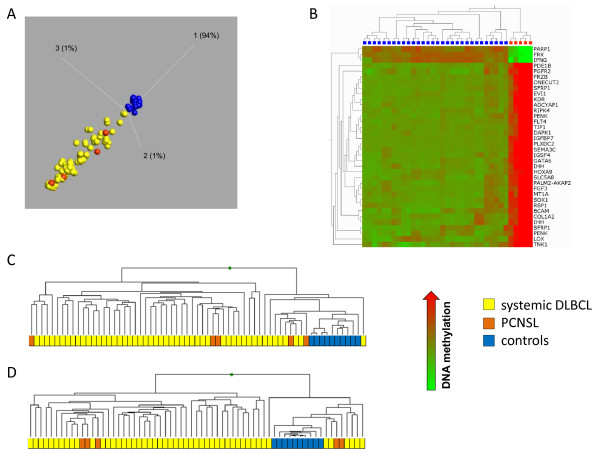
**DNA methylation analyses in PCNSL as compared to hematopoietic controls and systemic DLBCL**. (A) Principal Components Analysis (PCA) significantly separates control samples (blue circles) from PCNSL (orange circles) and systemic DLBCL (yellow circles) (p = 1.2 × 10^-12^; q = 3.9157 × 10^-11^) [40,41]. (B) Heatmap generated by applying the same conditions as in (A) red: high methylation level, green: low methylation level; blue squares: hematopoietic controls, orange squares: PCNSL; Hematopoieteic controls have been analysed in replicates and are shown as individual samples demonstrating good reproducibility (C) Hierachical cluster analysis of DNA methylation data obtained from the 296 CpG loci (corresponding to 194 genes) differentially methylated between five PCNSL (orange) and 10 hematopoietic controls (blue) including 49 systemic DLBCL (yellow boxes). CpGs differentially methylated in PCNSL and normal controls did not distinguish between PCNSL and systemic DLBCL. (D) Unsupervised hierarchical cluster analysis of methylation values of all 1,284 CpGs from 10 hematopoietic controls (blue boxes), five PCNSL (orange boxes) and 49 systemic DLBCL (yellow boxes). While normal and malignant samples were delineated according to their DNA methylation pattern, PCNSL and systemic DLBCL did not segregate.

PCA based analysis applying most stringent conditions differentiates normal controls from both PCNSL and systemic DLBCL samples in a highly significant fashion (p = 1.2 × 10^-12^, q = 3.9157 × 10^-11^; Fig. 1A). 33 CpG loci (corresponding to 30 genes) differentiating between PCNSL and controls performed in replicates were significantly hypermethylated while three CpG loci showed hypomethylation in PCNSL compared to hematopoietic controls (Fig. 1B).

Supervised cluster analysis of the 1,284 CpGs comparing the five PCNSL to ten hematopoietic controls resulted in 296 differentially methylated CpGs corresponding to 194 genes (Additional file 3). Of these 194 genes, 153 genes were hypermethylated while 41 genes were hypomethylated in PCNSL. Unsupervised cluster analysis of the five PCNSL and the 10 hematopoietic controls along with the 49 systemic DLBCL samples for all 1,284 CpGs separated all lymphoma samples from normal controls (Fig. 1C). These findings suggest that the methylation profile of PCNSL strongly differs from that of normal hematopoietic tissues including tonsillar germinal center B-cells and lymphoblastoid cells.

### CpG loci differentially methylated in PCNSL and systemic DLBCL

The methylation pattern of both PCNSL and systemic DLBCL were very heterogeneous with respect to total DNA methylation (data not shown). To address the question whether the DNA methylation pattern of PCNSL differs from that of systemic DLBCL, we compared DNA methylation values of 1,284 CpG loci from the five PCNSL with those of the 49 systemic DLBCL. Of the 194 genes differentially methylated between normal controls and PCNSL, using the same analysis approach, 157 genes were among the 174 genes differentially methylated between controls and DLBCL (Additional file 4). Neither supervised nor unsupervised cluster analysis separated PCNSL from systemic DLBCL (Fig. 1C, D, and data not shown). A differential methylation analysis (DMA) of PCNSL and systemic DLBCL cases yielded only four CpGs differentially methylated between PCNSL and systemic DLBCL (*ESR1, EFNA1*, *MATK*, and *PDE1B*). However, hierarchical cluster analysis performed on these four CpG loci failed to distinguish PCNSL from systemic DLBCL (Additional file 5). Thus, methylation analysis of these CpG loci does not allow a proper assignment of unknown samples to either PCNSL or systemic DLBCL. Furthermore, PCA failed to differentiate between PCNSL and systemic DLBCL (Fig. 1A, and data not shown). In conclusion, PCNSL did not exhibit a specific DNA methylation signature as compared to systemic DLBCL including more than 700 genes which had entered this study.

### Classification of genes based on their DNA methylation pattern in PCNSL as compared to non-neoplastic hematopoietic tissues

To get further insight into DNA hypermethylation in PCNSL, we compared the class of genes methylated in PCNSL but not in controls (meP/umC, n = 138) to the classes of genes unmethylated or methylated in both PCNSL and controls (umP/umC, n = 348 genes and meP/meC, n = 91 genes), respectively. These groups provided the basis for further analysis (Additional file 6).

### The group of genes methylated in PCNSL but unmethylated in non-neoplastic hematopoietic controls is enriched for polycomb targets in embryonic stem cells and promoters with high CpG content

**Figure 2:**
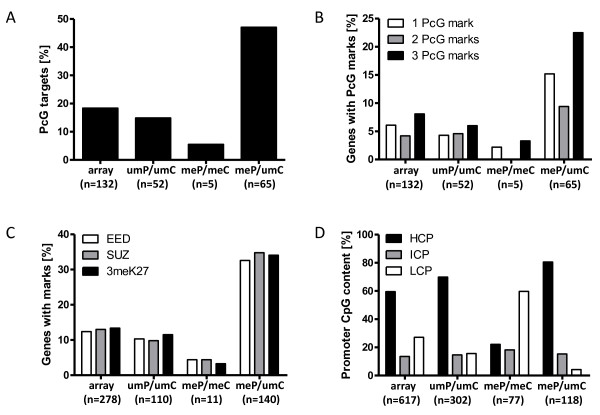
**Enrichment of PcG marks and HCP promotors in the group of genes methylated in PCNSL but unmethylated in non-neoplastic hematopoietic tissues**. Bar plot of the different DNA methylation subsets showing percentage and absolute numbers (numbers below each diagram) of (A) genes containing PcG marks in ESCs and (B) genes with one, two or three PcG marks, respectively. The meP/umC group was significantly enriched for PcG target genes (p < 0.0001; Fisher's exact test) and genes containing all three PcG marks (p < 0.0001). "array" reflects the distribution of the respective genes on the GoldenGate Cancer Panel I array. (C) Comparison of the content of EED, SUZ12 and 3 mK27-H3 target genes in the different groups and the GoldenGate array. The content of genes containing the individual marks is comparable in PCNSL. (D) Bar plot showing the percentage of promoter subtypes according to their CpG content in the different DNA methylation subsets. Genes de novo methylated in PCNSL (meP/umC) predominantly had promoters with high CpG content (p < 0.0001). In contrast, genes having promoters with low CpG content were enriched in the group showing high methylation in PCNSL and normal controls (meL/meC; p < 0.0001).

The group of genes unmethylated in the hematopoietic controls but methylated in PCNSL was highly significantly enriched for genes repressed by the polycomb (PcG) repressing complexes (PRC2) in embryonic stem cells compared to the genes present on the array (Fig. 2A; RR = 2.55; p < 0.0001, Fisher's exact test). This group was also enriched for the simultaneous presence of all three PRC2 marks as described previously [12,15,16]: EED (RR = 2.62), SUZ12 (RR = 2.68) and 3 mK27-H3 (RR = 2.54) (Fig. 2B). However, the targets of a specific PRC2 mark were not preferentially enriched in any group (Fig. 2C). While the percentage of PcG target genes in the group of genes unmethylated (um) in both controls (C) and PCNSL (P) (umP/umC) did not differ significantly from the one of the GoldenGate array (RR = 0.81; p = 0.1675), the percentage of PcG target genes was significantly reduced (RR = 0,30; p = 0.0010) in the group of genes methylated (me) in both PCNSL and normal controls (meP/meC) (Fig. 2A, B).

Since it was reported previously that particular promoters with high CpG content and CpG islands become methylated in tumors [19-22], we have sorted the genes in the groups defined above in genes having promoters with high (HCP), intermediate (ICP), or low (LCP) CpG content [12], respectively. While the group of genes methylated in both PCNSL and controls (meP/meC) was enriched for genes with low CpG content promoters (LCP; RR = 2.21; p < 0.0001), the remaining two groups were enriched by genes having promoters with high CpG content (umP/umC: RR = 1.17; p = 0.0023; meP/umC: RR = 1.35; p < 0.0001; Fig. 2D). The CpG loci belonging to genes methylated in PCNSL and controls were furthermore significantly enriched for loci located outside of CpG Islands (RR = 2.24; p < 0.0001).

### The group of genes differentially methylated in PCNSL is enriched for genes involved in neurological processes and cellular signaling pathways

As genes studied with the methylation-specific BeadArray were selected for their involvement in tumorigenesis, they will "by definition" be deregulated in various tumors. Even considering this bias, we tested whether genes differentially methylated in PCNSL and normal controls were enriched for specific Gene Ontology (GO) terms. Surprisingly, we observed that terms particularly involved in neurophysiological and perception processes were significantly enriched (Additional file 7). However, the same GO terms were also enriched in systemic DLBCL. Thus, a PCNSL specific enrichment of individual GO terms differing from DLBCL was not identified.

## Discussion

In the present study, we determined DNA methylation patterns in tumor samples of five patients suffering from PCNSL and compared them to the methylation pattern of 49 systemic DLBCL and ten normal hematopoietic controls, which have been characterized in detail before [12,14]. The GoldenGate Cancer Panel I used in this study offers the possibility to analyze 1,505 single CpG loci corresponding to 807 genes in parallel. To prevent any bias because of gender specific DNA methylation and imprinting effects, X-chromosomal genes and genes known to be imprinted, the methylation extent of which varies under normal physiological conditions, have been excluded from further analyses. The reproducibility and the accuracy of this array based approach have been extensively demonstrated before [12,18].

Of the 1,284 CpG loci focused on in this study, we identified 296 CpG loci to be differentially methylated between normal hematopoietic controls and PCNSL corresponding to 194 genes. These include numerous genes known to affect tumorigenesis e.g. by influencing cell proliferation (e.g. *CCNA1*), signal transduction (e.g. *JAK3*, *IGF1*, *FGF3*, *FGFR2*, *EGF*, *EGFR*), differentiation (e.g. *SOX1*, *SOX17*, *SOX2*, *MYOD1*, *PAX6*, *HOXA9*, *HOXB13*, *ETS2*) or spread of tumor cells within the brain (e.g. *MMP19*, *MMP2*, *MMP7*) [23-26]. Furthermore, by applying a more stringent PCA based approach, 36 CpG loci sufficient to differentiate between PCNSL and hematopoietic controls in a highly significant fashion have been identified.

Previous studies on DNA methylation in PCNSL were limited by their restriction to a low number of selected genes. Our data are in line with Chu et al. and Gonzales-Gomez et al. [5,8] analyzing the DNA methylation of *CDKN2B*, *DAPK1*, *GSTP1*, *MGMT*, *MLH1*, *RARB*, *THBS1*, *TIMP2*, and *TIMP3 *in PCNSL by MSP (Additional file 8) further supporting the validity of our analysis. This is of special interest, since our data on PCNSL are based on a minor number of five cases, because of the limited availability of sample material. Our results regarding *CDKN2A *are somewhat contradictory, however, one should keep in mind that the CpG loci analyzed by the GoldenGate array and by MSP are not identical. In addition, MSP data are not quantitative [27] and analysis of the *CDKN2A *locus might be affected by recurrent deletions of this gene in PCNSL [28].

A further analysis of genes which were unmethylated in hematopoietic controls and methylated in PCNSL (meP/umC) showed a significant enrichment of genes which are repressed by components of the PRC2 in embryonic stem cells. Components of the PRC2 complex are essential for the maintenance of the undifferentiated state of embryonic stem cells by suppressing genes called PcG target genes, the activation of which leads to cellular differentiation [16,29]. Indeed, hypermethylation of PcG target genes has been described for several tumor entities [30] including systemic mature aggressive B-cell lymphomas [12]. This enrichment of genes controlled by PRC2 during stem cell maintenance could lead to the hypothesis that the tumor cells of PCNSL cells derive from a stem cell like progenitor cell. Alternatively, dedifferentiation and epigenetic reprogramming of an already differentiated precursor cell could also explain the DNA methylation pattern [12]. The latter model is supported by previous data lending support for the hypothesis that the tumor cells derived from mature GC exit B-cells [3]. Finally, genes anyhow silenced in the hematopoietic lineage could become switched off by DNA methylation in tumor cells [12].

Interestingly, genes with three PcG-marks in the promoter region were significantly enriched, while there was no enrichment of a specific mark (neither EED, SUZ12 or 3 meK27-H3) in PCNSL. This is in line with results obtained for systemic mature B-cell lymphomas [12]. Furthermore, also similar to the scenario described for other B-cell lymphomas, in particular genes with high CpG content promoters become methylated in PCNSL. This correlates well with the known phenomenon that CpG islands in promoter regions become methylated during tumorigenesis [31-33].

In a recent study addressing genomic imbalances in PCNSL [34] several genes hypermethylated in PCNSL, including e.g. *ERBB3*, *PDE1B*, *ASCL1*, and *BCAM *were located in regions with recurrent genomic gains. However, they were not expressed in the tumor [3]. Since DNA methylation has been associated with gene repression in numerous studies [35-37], our data on DNA methylation might offer a putative explanation why increased gene dosage does not lead to increased gene expression and even transcriptional silencing in PCNSL. However, since a number of recent studies have shown little correlation between tumor specific hypermethylation and changes in gene expression [12,38,39], this must be addressed by future studies.

## Conclusions

Comparing the DNA methylation state of 1,284 CpG loci in PCNSL, systemic DLBCL, and hematopoietic controls, a DNA methylation pattern exclusively specific for PCNSL was not identified. The methylation data do not allow distinguishing PCNSL from systemic DLBCL on the basis of DNA methylation levels. However, the present study does not exclude the possibility that analysis of a larger number of CpG loci of the genome in a larger series of PCNSL might identify PCNSL specific features.

## Competing interests

MB and EG are employees of Illumina Inc.; JIMS and OA received speaker's honorary from Illumina Inc.; the study was performed in the framework of an R&D contract between Illumina Inc. and the group of RS.

## Authors' contributions

RS, MD, MB, ODW and JIMS designed the study; MMR and MD performed neuropathology and provided DNA; JR, JIMS, MB and EG performed the experiments, OA and JIMS performed data analysis; JR, OA, MD and RS wrote the manuscript, all authors approved the manuscript.

## Pre-publication history

The pre-publication history for this paper can be accessed here:

http://www.biomedcentral.com/1471-2407/9/455/prepub

## Supplementary Material

Additional file 1 **Table of DNA-methylation data (beta-values) obtained from the microarray analysis (0: unmethylated; 1: fully methylated).** Additionally, this table contains information about the sequence analyzed, the promoter class and whether the gene is a putative PcG target gene. Click here for file

Additional file 2 DNA samples used as controls in the present study (according to Hummel et al., 2006). Click here for file

Additional file 3 List of genes differentially methylated between controls, PCNSL and DLBCL Click here for file

Additional file 4 **GeneVenn Diagramm http://www.bioinformatics.org/gvenn/ of genes differentially methylated between 5 samples of PCNSL and 10 hematopoietic controls (control-PCNSL, red circle), between 49 cases of systemic DLBCL and 10 haematopoietic controls (control-DLBCL, yellow circle) or between 5 cases of PCNSL and 49 cases of systemic DLBCL (PCNSL-DLBCL, green circle), respectively.** There is a significant overlap of genes differentially methylated in both malignancies compared to controls. A detailed list of genes is presented in Additional file 3. Click here for file

Additional file 5 **Hierarchical cluster analysis of DNA methylation data obtained from 4 differentially methylated CpG loci in 5 cases of PCNSL (orange boxes in the bar blot below the dendrogramm) and 49 cases of systemic DLBCL (yellow boxes).** PCNSL and systemic DLBCL samples were not delineated according to their DNA methylation pattern. Click here for file

Additional file 6 List of genes unmethylated in normal controls and methylated in PCNSL (meP/umC), unmethylated in normal controls and unmethylated in PCNSL (umP/umC) or methylated in normal controls and methylated in PCNSL (meP/meC). Click here for file

Additional file 7 GeneOntology terms of genes differentially methylated in PCNSL (vs. controls) and DLBCL (vs. controls). RR, OR and p-value are shown. Click here for file

Additional file 8 **Comparision of the methylation frequency [%] of genes previously determined by other authors (Chu et al., 2006; Gonzalez-Gomez et al., 2003) using MSP and this study.** To calculate frequency based on the date obtained from the GoldenGate array, AVG-beta values above 0.3 where considered as methylated. Click here for file
