# Supplementary material for: Array-based DNA methylation profiling of primary lymphomas of the central nervous system
Source: BMC Cancer. 2009 Dec 21;9:455. doi: 10.1186/1471-2407-9-455 (PMC2807878; doi:10.1186/1471-2407-9-455)
Supplement: Additional file 2 — DNA samples used as controls in the present study (according to Hummel et al., 2006). [file 1471-2407-9-455-S2.DOC]

**Additional file 2.** DNA samples used as controls in the present study (Hummel et al., 2006).

LCL: lymphoblastoid B-cell line, PB: peripheral blood, n.a.: not available.

| Designation | Provider | Source | Age (years) | Gender |
| --- | --- | --- | --- | --- |
| Tonsil 1* | Institute for Cell Biology, Essen | Tonsil | 4 | male |
| Tonsil 2* | Institute for Cell Biology, Essen | Tonsil | 18 | male |
| NA06816 | Coriell Institute, Camden, USA | LCL | n.a. | female |
| NA06999 | Coriell Institute, Camden, USA | LCL | 15 | female |
| NA07033 | Coriell Institute, Camden, USA | LCL | 20 | male |
| NA10923 | Coriell Institute, Camden, USA | LCL | 39 | male |
| NA10924 | Coriell Institute, Camden, USA | LCL | 39 | female |
| NA14667 | Coriell Institute, Camden, USA | LCL | 45 | male |
| D32 | Institute of Human Genetics, Kiel | PB | 40 | female |
| D38 | Institute of Human Genetics, Kiel | PB | 44 | male |

* For the isolation of germinal center B-cells (Tonsil 1 and Tonsil 2), first CD19+ B-cells were enriched from tonsillar mononuclear cells by CD19-MACS (Miltenyi Biotech, Bergisch Gladbach, Germany). Germinal center B-cells were isolated from these B-cells by FACS as CD20high/CD38+ cells, using CD20-FITC and CD38-APC (both from Becton Dickinson, Heidelberg, Germany). Cell purity was 98%.
