# Supplementary material for: Array-based DNA methylation profiling of primary lymphomas of the central nervous system
Source: BMC Cancer. 2009 Dec 21;9:455. doi: 10.1186/1471-2407-9-455 (PMC2807878; doi:10.1186/1471-2407-9-455)
Supplement: Additional file 4 — GeneVenn Diagramm http://www.bioinformatics.org/gvenn/ of genes differentially methylated between 5 samples of PCNSL and 10 hematopoietic controls (control-PCNSL, red circle), between 49 cases of systemic DLBCL and 10 haematopoietic controls (control-DLBCL, yellow circle) or between 5 cases of PCNSL and 49 cases of systemic DLBCL (PCNSL-DLBCL, green circle), respectively. There is a significant overlap of genes differentially methylated in both malignancies compared to controls. A detailed list of genes is presented in Additional file 3. [file 1471-2407-9-455-S4.PPT]

## Slide 1
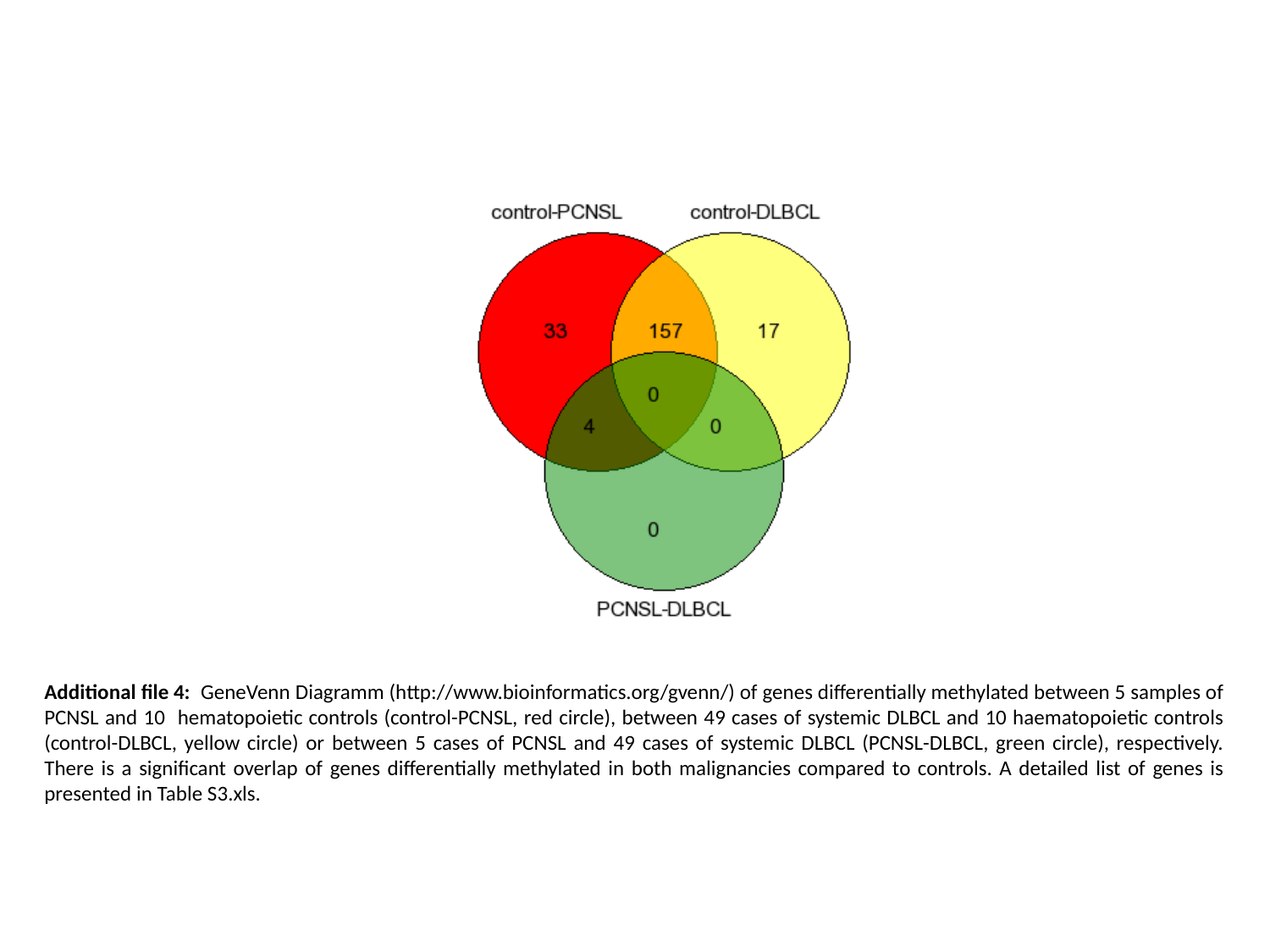

Additional file 4: GeneVenn Diagramm (http://www.bioinformatics.org/gvenn/) of genes differentially methylated between 5 samples of PCNSL and 10 hematopoietic controls (control-PCNSL, red circle), between 49 cases of systemic DLBCL and 10 haematopoietic controls (control-DLBCL, yellow circle) or between 5 cases of PCNSL and 49 cases of systemic DLBCL (PCNSL-DLBCL, green circle), respectively. There is a significant overlap of genes differentially methylated in both malignancies compared to controls. A detailed list of genes is presented in Table S3.xls.
