# Supplementary material for: Array-based DNA methylation profiling of primary lymphomas of the central nervous system
Source: BMC Cancer. 2009 Dec 21;9:455. doi: 10.1186/1471-2407-9-455 (PMC2807878; doi:10.1186/1471-2407-9-455)
Supplement: Additional file 5 — Hierarchical cluster analysis of DNA methylation data obtained from 4 differentially methylated CpG loci in 5 cases of PCNSL (orange boxes in the bar blot below the dendrogramm) and 49 cases of systemic DLBCL (yellow boxes). PCNSL and systemic DLBCL samples were not delineated according to their DNA methylation pattern. [file 1471-2407-9-455-S5.PPT]

## Slide 1
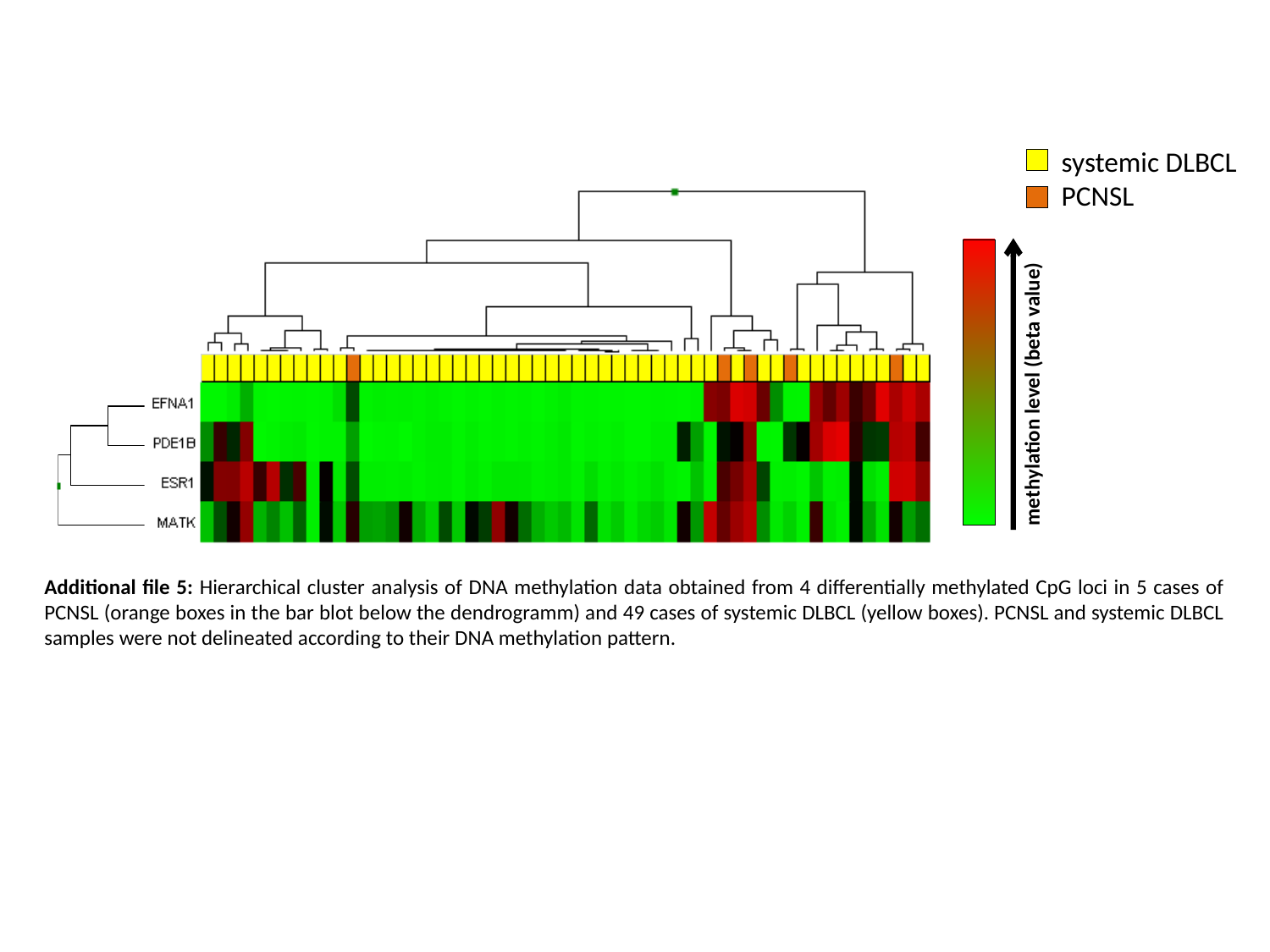

systemic DLBCL
PCNSL
methylation level (beta value)
Additional file 5: Hierarchical cluster analysis of DNA methylation data obtained from 4 differentially methylated CpG loci in 5 cases of PCNSL (orange boxes in the bar blot below the dendrogramm) and 49 cases of systemic DLBCL (yellow boxes). PCNSL and systemic DLBCL samples were not delineated according to their DNA methylation pattern.
