# Supplementary material for: Array-based DNA methylation profiling of primary lymphomas of the central nervous system
Source: BMC Cancer. 2009 Dec 21;9:455. doi: 10.1186/1471-2407-9-455 (PMC2807878; doi:10.1186/1471-2407-9-455)
Supplement: Additional file 6 — List of genes unmethylated in normal controls and methylated in PCNSL (meP/umC), unmethylated in normal controls and unmethylated in PCNSL (umP/umC) or methylated in normal controls and methylated in PCNSL (meP/meC). [file 1471-2407-9-455-S6.DOC]

**Definition of Gene Groups**

(Additional file 6, Richter et al.)

Genes unmethylated in normal controls and methylated in PCNSL (meP/umC):

ABO, ACVR1C, ADAMTS12, ADCYAP1, AGTR1, ALK, APP, ASCL1, BCAM, BMP2, BMP4, CALCA, CAV2, CCKBR, CCNA1, CDH1, CDH11, CDH3, CEBPA, CHFR, CHGA, COL18A1, COL1A2, CSF1, CSPG2, CTSL, DAB2, DAPK1, DCC, DES, DST, EFNA1, EGFR, EPHA3, EPHA5, EPHA7, EPHB3, EPHB4, EPO, ERBB3, ERBB4, ESR1, ETS2, ETV1, EVI1, EYA4, FGF3, FGF5, FLT1, FLT3, FLT4, FRZB, FZD7, GALR1, GAS7, GATA6, GDF10, GJB2, GNMT, GUCY2D, HHIP, HIC2, HLF, HOXA9, HOXB13, HS3ST2, HTR1B, ICA1, IGF1R, IGFBP3, IGFBP5, IGFBP7, IGSF4, IGSF4C, IHH, IL11, INHA, INSR, IRAK3, ISL1, KDR, KIT, LMO1, LOX, MATK, MLF1, MME, MMP2, MT1A, MYH11, MYLK, MYOD1, NGFB, NOTCH3, NPY, NTRK2, NTRK3, NTSR1, ONECUT2, OPCML, PALM2-AKAP2, PAX6, PDE1B, PDGFRA, PENK, PLXDC2, PRKCDBP, PROK2, PTGS2, RAB32, RARB, RARRES1, RBP1, RIPK4, ROR1, ROR2, SCGB3A1, SEMA3C, SFRP1, SLC5A8, SLIT2, SOX1, SOX17, SOX2, TAL1, TBX1, TCF7L2, TFAP2C, TFPI2, THBS1, TIMP2, TJP1, TMEFF2, TNFRSF10C, TWIST1, WNT2, ZMYND10, ZP3

Genes unmethylated in normal controls and unmethylated in PCNSL (umP/umC):

ABCA1, ABCC5, ABCG2, ABL1, ABL2, ACVR1B, ACVR2B, ADAMTS12, AFF3, AHR, AKT1, ALPL, APC, APP, AREG, ARNT, BCAM, BCL2L2, BCL3, BCL6, BIRC5, BMP3, BMP6, BMPR1A, BMPR2, BSG, CAPG, CASP10, CASP2, CASP3, CASP6, CASP8, CAV1, CCL3, CCNC, CCND1, CCND2, CCND3, CCNE1, CD2, CD34, CD40, CD44, CD82, CD9, CDC25B, CDH3, CDK10, CDK2, CDK6, CDKN1A, CDKN1B, CDKN2A, CDKN2B, CEACAM1, CFTR, CHFR, CLK1, COL1A1, COL4A3, COMT, CPNE1, CREB1, CRIP1, CSK, CSTB, CTNNA1, CTNNB1, CTSH, CTTN, DAB2IP, DDB2, DDR1, DKFZP564O0823, DLL1, DNAJC15, DNMT1, DNMT3B, DSP, DUSP4, E2F3, E2F5, EDN1, EFNA1, EFNB3, EGR4, EIF2AK2, ELK3, EMR3, EMR3, ENC1, EPHA1, EPHB1, EPHB2, EPHB6, EPM2A, EPS8, ERBB2, ERCC1, ESR2, ETS1, ETV6, EVI2A, EXT1, F2R, FANCE, FANCF, FANCG, FAS, FER, FES, FGF8, FGF9, FGFR1, FGFR3, FHIT, FLI1, FLT4, FN1, FVT1, FYN, FZD9, GAS1, GJB2, GPX1, GPX3, GSTM2, GSTP1, GUCY2D, HBEGF, HCK, HDAC1, HDAC11, HDAC9, HFE, HIC1, HIF1A, HLA-DPA1, HLA-DPB1, HLA-DRA, HLA-F, HOXA9, HOXC6, HPN, HPSE, HSD17B12, ICAM1, ID1, IFNGR1, IFNGR2, IGF1R, IGFBP5, IGFBP6, IL10, IL11, IL12A, IL17RB, IL18BP, IL6, IL8, IRF5, IRF7, ITGA2, ITGA6, ITGB1, ITGB4, ITPR2, ITPR3, JAG1, JAG2, JAK2, JAK3, JAK3, JUNB, KIAA1804, KLF5, KLK10, KRAS, LAMC1, LAT, LIF, LIG4, LMO1, LRP2, LRRC32, LTA, LYN, MAF, MALT1, MAP2K6, MAP3K1, MAP3K9, MAPK12, MAPK14, MCAM, MCC, MCM2, MCM6, MGMT, MLH1, MLH3, MLLT3, MLLT6, MMP2, MMP7, MMP9, MPL, MPO, MST1R, MTA1, MUC1, MXI1, MYB, MYBL2, MYCL1, MYCN, MYH11, NCL, NEU1, NFKB1, NFKB2, NGFR, NKX3-1, NOTCH1, NOTCH2, NOTCH4, NPY, NQO1, NR2F6, NRAS, NTSR1, OAT, ODC1, OPCML, OSM, P2RX7, PADI4, PAX6, PCDH1, PCGF4, PDGFA, PDGFRB, PGF, PITX2, PKD2, PLA2G2A, PLAU, PLAUR, PLXDC1, POMC, PPARD, PPARG, PPP2R1B, PRKAR1A, PRKCDBP, PSCA, PSIP1, PTCH, PTEN, PTGS1, PTHLH, PTHR1, PTK2, PTK2B, PTPN6, PTPNS1, PTPRF, PTPRG, PTPRH, PTPRO, PURA, PXN, PYCARD, RAD54B, RAF1, RAP1A, RARA, RASA1, RASSF1, RBL2, RET, RHOC, RIPK2, RIPK3, RRAS, RUNX1T1, RUNX3, RYK, SEMA3B, SEMA3F, SEZ6L, SFN, SH3BP2, SHB, SKI, SMAD2, SMAD4, SMARCA3, SMARCA4, SMO, SPARC, SPI1, SPP1, ST6GAL1, STAT5A, STK11, SYK, TCF4, TDGF1, TERT, TESK2, TFRC, TGFA, TGFB2, TGFBR3, THBS2, TIAM1, TIMP2, TJP2, TK1, TMEFF1, TMEM63A, TNF, TNFRSF10A, TNFRSF10B, TNFRSF10D, TNFRSF1B, TNFSF10, TNFSF8, TRAF4, TRIP6, TSG101, TUBB3, TYK2, TYRO3, UBA52, UNG, VAMP8, VAV1, VAV2, VEGFB, VIM, WNT1, WNT10B, WNT2B, WNT5A, WRN, YES1

Genes methylated in normal controls and methylated in PCNSL (meP/meC):

AATK, ABCB4, ABCC2, ACVR1, AFF3, AFP, AGXT, ARHGAP9, BCL2A1, BRCA1, CD1A, CDH17, CHD2, CREBBP, CTGF, CYP2E1, DNMT2, DSC2, DSG1, EDNRB, EGF, EPHA8, FANCA, FER, FGF6, FGF7, FOLR1, GLI2, GML, GNG7, GPR116, GSTM1, HGF, HLA-DOB, IL13, IL1A, IL3, ITGA6, ITK, KIAA0125, KRT5, LCN2, LRRK1, LY6G6E, MAD2L1, MAPK10, MAPK9, MAS1, MC2R, MMP1, MMP19, MMP3, MSH2, MSH3, MST1R, NID1, NKX3-1, NPR2, PGR, PI3, PIK3R1, PMP22, PRSS8, PTHR1, PTPRH, RARRES1, RIPK1, S100A12, S100A4, SERPINB2, SERPINB5, SFN, SFTPA1, SIN3B, SNCG, SRC, TEK, TGFB1, TGFB3, TIMP3, TMPRSS4, TRIP6, TSP50, UGT1A1, UGT1A7, WNT8B, WRN, XRCC1, XRCC2, ZNFN1A1, ZP3
