# Supplementary material for: Array-based DNA methylation profiling of primary lymphomas of the central nervous system
Source: BMC Cancer. 2009 Dec 21;9:455. doi: 10.1186/1471-2407-9-455 (PMC2807878; doi:10.1186/1471-2407-9-455)
Supplement: Additional file 8 — Comparision of the methylation frequency [%] of genes previously determined by other authors (Chu et al., 2006; Gonzalez-Gomez et al., 2003) using MSP and this study. To calculate frequency based on the date obtained from the GoldenGate array, AVG-beta values above 0.3 where considered as methylated. [file 1471-2407-9-455-S8.PPT]

## Slide 1
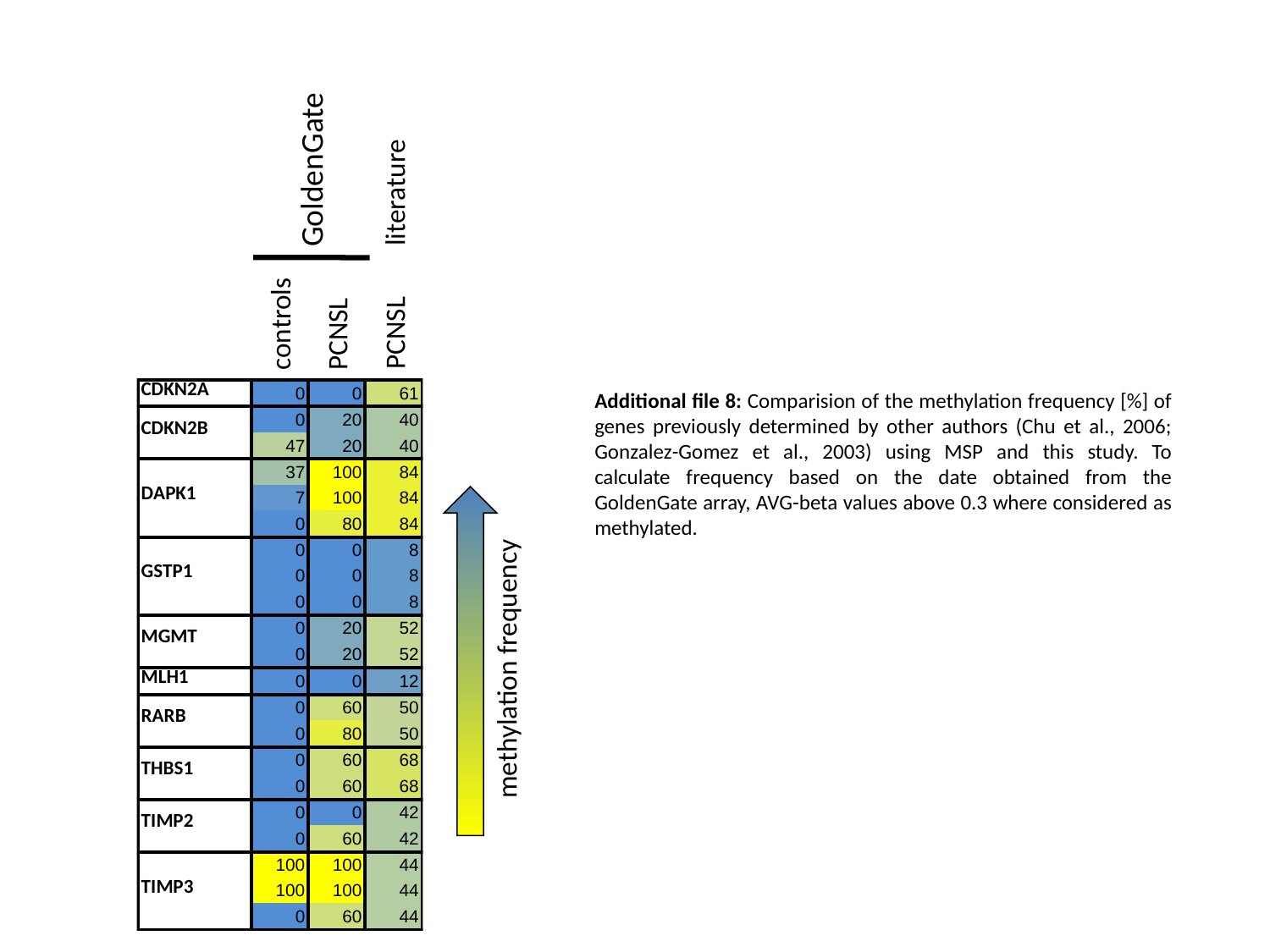

GoldenGate
PCNSL literature
controls
PCNSL
Additional file 8: Comparision of the methylation frequency [%] of genes previously determined by other authors (Chu et al., 2006; Gonzalez-Gomez et al., 2003) using MSP and this study. To calculate frequency based on the date obtained from the GoldenGate array, AVG-beta values above 0.3 where considered as methylated.
methylation frequency
